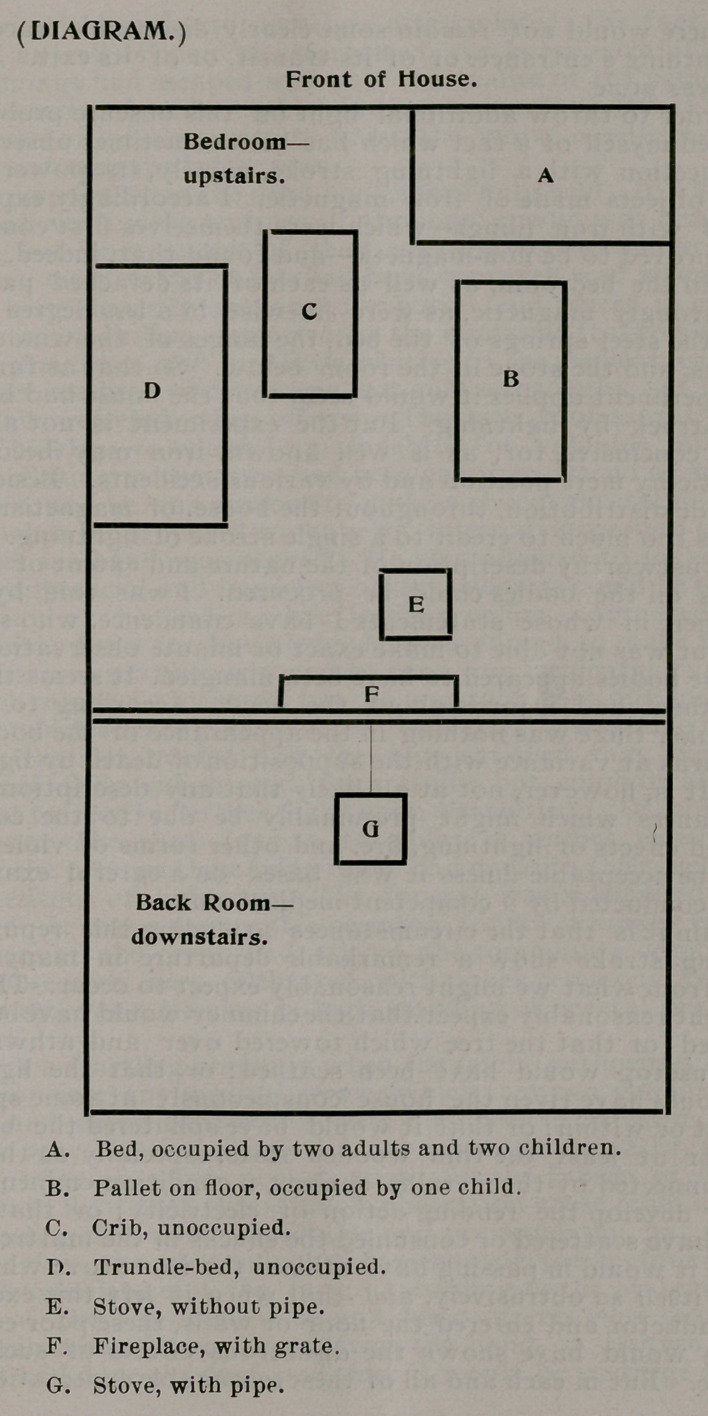# Death by Lightning

**Published:** 1897-07

**Authors:** William H. Taylor

**Affiliations:** Professor of General Chemistry, Toxicology, and Medical Jurisprudence in the Medical College of Virginia


### DEATH BY LIGHTNING.

By WILLIAM H. TAYLOR, M. D.

Professor of General Chemistry, Toxicology, and Medical Jurisprudence in the Medical College of Virginia.

There is a very general belief that the annual number of deaths by lightning is increasing. Statistics appear to prove that this is so, and it is quite likely that some of the changes in physical and social conditions incident to the novel and varied activities of our time may tend to multiply electrical storms and enhance their dangers. But however it may be in Europe, where the reality of this increase has been specially insisted upon, or in other parts of our own country, I am of opinion that it is not true of this locality. At any rate, my personal knowledge of events in Richmond, where I have resided all my life, and my experience as its coroner for the past twenty-five years, convince me that the risk of death by lightning in this city is very small indeed.

One afternoon, some fifty years ago, two young ladies, while together in a house on Church Hill, in Richmond, were killed by a lightning stroke. The impression which was made upon the minds of the people by this catastrophe was intense and lasting. The memory of it has been passed down to our own days, all of its circumstances are still well known, and is still talked about as a notable and deeply interesting incident of our local history. Between the time of this occurrence and 1872, when I became coroner of Richmond, I know of no instance of death by lightning in this city. It is, however, of course possible that in the interval deaths may have been occasioned by this cause and not have come to my knowledge.

All cases of death by lightning are properly subjects of inquiry by a coroner and should be reported to him. During my twenty-five years of service only two cases have been brought to my notice. I have heard of two other deaths during this period, one of a man and one of a child, which were attributed to lightning, but whether justly or not I can not say. But, taking these into account, it appears that in twenty-five years there have been at the most only four deaths by lightning in Richmond—which is certainly not a formidable proportion for a population which has ranged from 55,000 to 85,000. In fact, as a menace to life, artificial electricity is much more to be feared than natural electricity, for the former during the nine years of its general use in this city has directly or indirectly killed sixteen persons.

The first of the two cases of death by lightning which have come under my notice occurred on the afternoon of June 8, 1881. A negro man was on a sandbar, a few yards from the river at the south end of Seventeenth street, engaged in shoveling sand into a mule-cart, when he was struck and instantly killed. The bolt came near meriting the description of a flash of lightning from a clear sky, for there was no rain, and there were no clouds except a small and mild-looking bank in the west, nor had there been any thunder before the peal which accompanied the fatal stroke; in fact, the whole display was exceedingly unimpressive. Two other persons who were standing near escaped altogether. Whether the mule was struck is not known, but, at any rate, he suffered a degree of discomposure, being moved to depart from the place, and he did so in some haste, kicking the cart up into the air as he left. In the dock, close by, were several vessels, and it is somewhat remarkable that the lightning did not select their high masts rather than so low an object as the man.

On inspection there was found an excoriation on the right side of the forehead at its upper part, and at the junction of the left clavicle and sternum was another excoriation, from which a little blood had effused. Still other excoriations were on the left shoulder, while along the left arm were several spots where the cuticle was raised in vesicles. These were the only external injuries; they were individually small in size, and collectively were, in appearance at least, quite unimportant.

My second case was that of a white man who was instantly killed on the afternoon of June 18, 1896, at the very beginning of a storm, while he was at work with a shovel on a street at the extreme western end of the city. He was alone, but persons at a distance saw him fall. The place was a very open one, although Richmond College, which has a high tower, and several other buildings with rather tall chimneys were in the vicinity, and perhaps, near enough to render them liable to receive the stroke.

I saw this man about an hour and a quarter after his death. He was lying upon his back, and there was then no rigor mortis. I examined the body more fully when it had been dead about two hours; there was still no rigor, but cadaveric lividity had developed itself very strongly on the back. Whether rigor had already rapidly come and gone, as it is said to sometimes do after death by lightning, I could not ascertain. His countenance was placid, the mouth being slightly opened, and there was no froth or other discharge about the lips or nostrils. The eyes were closed and both pupils were widely dilated. There was a mark on the right temple of the nature of a vesiculation with its cuticle gone. It was situated about midway between the eye and ear, its lower end on a line with the orbital arch, and extended vertically upwards for one inch and a half, and it was three-eights of an inch in breadth. The hair on the chest was singed and frizzled, and the skin of the throat and that of nearly the whole chest was greatly reddened, perhaps by the heat from the burning hair. At a point close to the umbilicus began a narrow, faintly-red streak, and it continued in a straight line obliquely to the anterior superior spine of the right ilium, where it terminated in a great number of closely placed, small indentations, very much resembling marks made by the impingement of small shot. This singular display was of brownish-red color and of oval shape, about three inches long and one inch wide at its middle, and tapering towards its extremities. It was conjectured that the electric current left the body at this place, passing to the shovel which had been held in the right hand. There were, however, no appearances of injury to the hand. As was the case with the negro man, the body was not lacerated or otherwise seriously disfigured.

I greatly regret that I am not in a position to describe the internal condition of these two bodies; but, indeed, neither custom nor the law, as they prevail in Richmond, authorizes the coroner to make an autopsy in the pursuit of knowledge merely—there must be a question of criminality, real or supposed, to justify the procedure. And moreover, as my experiences in a case with which I was once concerned have taught me, to so refine a pitch of virtuous propriety have certain of our lawgivers attained, that they would have the operator, when the subject is a woman, do his nefarious work alone, at midnight, in a cellar, with his head in a bag. Wherefore, since I could offer no sufficient justification for the use of the knife and saw on either of these two bodies, I had to content myself with making only such observations as a decent respect for the opinions of my fellow-citizens permitted.

A remarkable instance of what was apparently killing by lightning occurred on the night of July 19, 1892, in a house situated a short distance beyond the limits of Richmond in the county of Henrico. During a violent storm a very loud peal of thunder was heard and a few minutes afterwards—at about half past nine o'clock—persons living in the neighborhood discovered that this house was on fire, and gave an alarm. When the firemen entered the building they found that the fire was in the upper room—there being but one upper room in the house—and that in this room were the dead bodies of the whole family, consisting of a man named Dale Emmett, a woman, and three children.

As this case had occurred out of Richmond I could not investigate it officially, but it was one which had for me much of scientific interest and I inquired into it as fully as circumstances allowed. I did not have the opportunity of inspecting the bodies, for when I visited the place, which was early on the following morning, they were gone—having, soon after the killing, been taken in charge by a magistrate of the county in his capacity of acting coroner, and consigned to the ruinous restorations of an undertaker. The room itself was in extreme confusion, the damage done to it being great and of varied character, and its state was such as made it impossible to determine accurately what part of the destruction should be apportioned to the fire and what to the firemen.

It was also difficult to obtain trustworthy accounts of the occurrence, for the people of the neighborhood were naturally much perturbed, and accordingly disposed to contemplate the affair chiefly in its marvelous and mystical aspects, and so were impelled to misstate and to magnify and to manufacture facts. A man to whom I applied for information, while regretfully admitting that he did indeed know but little of this particular visitation, nevertheless favored me with a most circumstantial and excellent description of another which he knew all about, which had descended some years ago upon this very spot, and which destroyed a tree near the house, slew his uncle, threw his aunt forty feet away, seized hold of a stream flowing hard by, tore it up by the roots, and gave it such a twist that it has run crooked to this day. On the other hand, I encountered a woman who assured me that she could tell me anything I could possibly want to know, for that she had seen the lightning jump down from the sky, pounce on top of the house, go through it, and then set out towards her own self, and that she had barely had time to step aside out of its way before it came rushing past her. However, persistent inquiry and carefully conducted observation of the locality enable me to give the following account, which, though incomplete, is, I believe, as far as it goes, substantially correct.

The house was a detached wooden building with three rooms, a lower and an upper one of the same size, and another lower one in rear of the first. The roof sloped from the four sides moderately upwards to a point. The chimney was in the middle between the two lower rooms and therefore passed through the back part of the upper room. The upper room was the bedroom, and, besides beds, it contained a stove, the pipe of which was missing, but whether it had been removed before, during, or after the fire is uncertain. This room was about fifteen feet square and of low pitch. The back lower room contained a stove with a pipe going into the chimney. Several trees, which were higher than the house, were near it, and one of these especially was so close to it and so tall that its limbs overshadowed the roof.

The fire had been confined to the bedroom, but when I first saw the room it was, as has been said, in great disorder. It and its contents had been severely treated in the energetic efforts made to save the inmates and to extinguish the flames. The roof of the house also had been much broken by the firemen. In the excitement of the time the position and attitude of the several bodies had not been closely noted, nor had the place of origin of the fire and its line of progress been observed. Statements concerning these particulars were conflicting, and it was not now possible to ascertain the facts very exactly. I was, however, able to construct the accompanying diagram, which may be taken to be a reasonably correct representation.

As the objects were many and some of them bulky, they must have filled the small room very compactly. The bedstead was of wood and stood on porcelain casters. The bed was a shuck mattress supported by a set of narrow and thin wooden slats which were attached to a corresponding under set by intervening stout steel springs. Through the mattress from top to bottom was an opening large enough to admit the hand. Possibly this opening was made by burning, for both its upper and under borders were browned, though not distinctly charred, but there was no burnt smell about it and the shucks were not in the least scorched. But, whatever may be the signification of this opening, I have reason to believe that, in fact, it was not made during the thunderstorm but afterwards —peradventure by some of the enthusiastic body of amateur investigators who flock to scenes like this, and in their zealous inquiry reduce everything to a state which is forever after beyond the reach of rational interpretation.

I proceeded to make a careful scrutiny of the room, and of the house, and of it surroundings, and was astonished to find that nowhere was there to be seen any unequivocal mark of a visitation of lightning. Making due allowance for the great obliterative powers of fire, and the still greater ones of an earnest salvage corps, it was, nevertheless, almost incredible

(DIAGRAM.)

A. Bed, occupied by two adults and two children.

B. Pallet on floor, occupied by one child.

C. Crib, unoccupied.

D. Trundle-bed, unoccupied.

E. Stove, without pipe.

F. Fireplace, with grate.

G. Stove, with pipe.

that there would not remain some clearly discernible trace of the lightning's entrance, or of its transit, or of its exit. But there was none.

In order to throw additional light on this obscure problem I availed myself of a fact which has been sometimes observed in connection with a lightning stroke, namely, its power to render objects made of iron magnetic. I accordingly experimented with iron filings—which were themselves first conclusively proved to be non-magnetic—and found that, indeed, the stove in the bedroom, as well as each of its detached parts, was strongly magnetic, as were likewise in a less degree the grate, the steel springs of the bed, the hinges of the window-shutters, and the stove in the room below. So that as far as this experiment applies it would seem that the house had been really struck by lightning. But the experiment is not altogether conclusive, for, as is well known, iron may become magnetic by mere position and by various accidents. Besides, this wide distribution, throughout the house, of magnetism is perhaps too much to credit to a single stroke of lightning.

No trustworthy description of the nature and extent of the injuries on the bodies could be procured. I was told by a gentlemen, in whose statements I have confidence, who saw them but was not able to make exact or minute observations, that the bodies appeared to have been mangled. It seems that all of them had injuries about the head. According to the undertaker there was nothing in the appearance of the bodies which was at variance with the supposition of death by lightning. It is, however, not at all likely that any description of appearances which might presumably be due to the compounded effects of lightning, fire, and other forms of violence would be acceptable unless it was based on a careful examination conducted by a competent medical man.

Certain it is that the circumstances attending this reputed lightning stroke show a remarkable departure in many respects from what we might reasonably expect to occur. Thus we might reasonably expect that the chimney would have been damaged; or that the tree which towered over and athwart the housetop would have been scathed; or that the lightning would have riven the house conspicuously at some spot without or within; or that it would have splintered the bedstead, or at least the thin wooden slats, especially as these were connected by the metallic springs, a condition eminently apt to develop the rending action of electricity; or that it would have scattered or consumed the shucks in the mattress; or that it would in passing have utilized the iron stove which offered itself so obtrusively, and that when it left this excellent conductor and entered the floor or walls, these poor conductors would have shown the disastrous effects of such a passage. But in each and all of these reasonable expectations we would have found ourselves disappointed; the fact being that, as well as could be ascertained, the house with all of its belongings had escaped and only the bodies of the persons in it had suffered—a fact which is most anomalous, for it is not the method of electricity to spare the worse conductors and scar and tear the better ones.

An inquest upon the dead bodies was duly held. It was conducted, not by the coroner of the county, who was a medical man, but by a justice of the peace, and at the undertaker's rooms, whither the bodies had been conveyed and where they had been decently prepared for burial and placed in coffins. Very much evidence relative to the electrical features of the case was taken, and very little relative to the medical features. In fact, medical evidence was not only not sought, but it was actually rejected when offered. The late Professor Charles H. Chalkley—to whom I am indebted for valuable information relating to the case—had been asked to attend as medical expert, and he was anxious to make a thorough examination, but when he proposed to have an internal inspection of the bodies his hand was stayed. He was allowed to make only such superficial and imperfect observations as could be had by looking at the bodies while in their coffins. Actuated by a laudable desire for knowledge he even offered to make the examinations gratuitously and to bear any incidental expenses which might be incurred, but he was told that any information which science might flatter itself that it was able to furnish was not needed, and the jury, satisfied that they were already fully enlightened, respectfully but emphatically declined to receive further light upon the subject. Of course it did not take them long to formulate a verdict, which was that the persons there lying dead had come to their death by the effects of lightning. The funeral was solemnized soon after. It was a memorable occasion, for the dramatic circumstances of the event had stirred the community deeply, and the obsequies were celebrated with much ceremony, for Mr. Emmett was a member in high standing of several organizations.

It is a startling anomaly when the public authorities fail to appropriate any medical service which they can get for nothing, and many conjectures were advanced in attempts to explain why Henrico county did not instantly clutch Dr. Chalkley's offer of a free post-mortem. In the fulness of time it came to light that this strange aberration arose from a state of feeling the most respectable and praiseworthy, but, at the same time, the most unlooked for—namely, the profound piety of the gentlemen of the jury. According to the statement of one of these gentlemen, he and his confreres were moved by the pious conviction that it was the hand of Heaven itself that had done the deed, and that it would be sacrilege should any human hand less reverent than an undertaker's disturb the consecrated victims further. The religious conception of the sanctifying power of lightning, which, it may be observed, has a most venerable antiquity to commend it, was supplemented in the minds of this saintly jury by an appeal to the best established principles of ratiocination; for, they reasoned, here was a house with every one in it dead and mangled, it was on fire, a terrible thunder-storm was prevailing; therefore it was the lightning which killed these persons—clearly, it could have been nothing else. And so the gentlemen of the jury, having theology and logic both to back them, brought in their verdict as aforesaid.

Mr. Emmett was a man of unblemished reputation, and held in high esteem. There was nothing to raise a doubt as to the alleged mode of death of him and his family, except in the minds of the few troublesome persons, found in every community, who obstinately refuse to understand things they do not comprehend, and sometimes even pass oblique reflections on the decision of a coroner's jury. These persons professed to think that, after liberally allowing for the admitted waywardness of electricity, there were at least some laws which it was bound to respect; but that in this case these laws had been grossly violated. Everybody else, however, saw a sufficient explanation of the anomalous features in that vast and authoritative body of knowledge known under the name of the "vagaries of lightning," as set forth from time immemorial by many Greek and Roman historians and philosophers, and by their own grandmothers and aunts. But, at the most, it was thought to be a mere speculative question, and so the matter was allowed to drift away among the recollections of the past.

And the matter might have drifted altogether out of mind but that not very long after Mr. Emmett had been so impressively laid to rest, the astounding discovery was made that he was a rogue, and an otherwise all-round scoundrel of the first class; that his wife and children were not his at all, but the property of another man, which he had stolen and run away with. This discovery of course put a different face upon the circumstances of the tragedy, and it was realized that the death of this family could be reasonably attributed to some other agency than lightning. The question, now become very prominent and very perplexing, could not be resolved decisively. It might, perhaps, have been so resolved had the permission to examine the dead bodies which Dr. Chalkley asked for been granted. But the jury of inquest, as we have seen, conducted their researches in a deeply religious spirit, and did their thinking according to the canons of good, hard, common sense; and a medical examination under these circumstances was out of place, superfluous, and had to be rejected.

In contemplating the attitude of these pious and discerning men towards this singular case, while we may feel ourselves constrained to applaud their eminent piety, and to admire the closeness of their reasoning, there yet appears to be room for the conjecture that had the former attribute been a trifle less over-sublimated, and the latter a little broader in its scope, we should not now find ourselves sometimes wondering if, after all, this may not have been a case of murder, arson, and suicide.—*University Medical College Bulletin.*

## Figures and Tables

**Figure f1:**